# Diurnal and nocturnal salivary fluoride bioavailability following brushing with high‐ or regular‐fluoride dentifrices

**DOI:** 10.1111/eos.70079

**Published:** 2026-03-04

**Authors:** Guereth Carvalho, Guilherme Santana, Niciane Macena, Gláuber Vale

**Affiliations:** ^1^ Department of Restorative Dentistry Federal University of Piaui Teresina, Piauí Brazil

**Keywords:** biological availability, circadian rhythm, fluoride treatment

## Abstract

This study evaluated salivary fluoride (F) bioavailability during diurnal and nocturnal periods after using a high‐fluoride or regular dentifrice. Fifteen healthy adults participated in a double‐blind crossover study, brushing with either a 1450 ppm F or 5000 ppm F dentifrice. Saliva was collected at pre‐brushing, immediately after brushing, and at 5 min, 2, 4, and 8 h post‐brushing in both periods. Fluoride concentrations were measured using an ion‐selective electrode and analyzed with the Wilcoxon matched‐pairs and Kruskal–Wallis tests (5% significance). At pre‐brushing, no statistically significant differences were detected between groups. Both dentifrices increased salivary fluoride concentrations, reaching peak levels immediately after brushing. However, the high‐fluoride dentifrice consistently maintained significantly higher concentrations compared with the regular formulation. During the nocturnal period, elevated fluoride levels persisted for up to 8 h with the high‐fluoride dentifrice, whereas concentrations returned to pre‐brushing levels within 2 h with the regular formulation. In conclusion, fluoride bioavailability was greater and more sustained during the nocturnal period than during the diurnal period, emphasizing the influence of brushing time on salivary fluoride retention. High‐fluoride dentifrice further enhanced this effect across both periods.

## INTRODUCTION

The use of fluoride (F) remains the most effective strategy for preventing and controlling dental caries at both individual and population levels [1]. Its mechanism of action is physicochemical, promoting enamel and dentin remineralization while reducing demineralization [2]. Among various fluoride delivery methods, dentifrices are considered the most rational approach, as they combine mechanical biofilm disruption with the topical availability of fluoride to support de‐ and remineralization processes [3, 4]. The widespread use of fluoride dentifrices has been identified as a major contributor to the global decline in dental caries in recent decades [5].

For fluoride to be effective, it must be free and soluble in the aqueous oral environment (saliva or biofilm fluid), and its anticaries activity therefore depends on its bioavailability in these fluids [1, 6]. Following topical fluoride application, such as toothbrushing with fluoridated dentifrice, salivary fluoride concentrations increase proportionally to the fluoride content of the product used [7]. Although salivary flow dilutes fluoride levels post‐brushing, fluoride can be retained in enamel, residual biofilm, and oral soft tissues (such as the tongue and mucosa) [3, 8, 9]. Fluoride stored in these reservoirs can later be released into the biofilm fluid, particularly during pH drops caused by sugar intake, thereby prolonging its protective effect.

The salivary bioavailability of fluoride after toothbrushing is limited to a relatively short period. While fluoride concentrations can exceed pre‐brushing levels by more than 100‐fold within the first 3 min after brushing, they typically return to pre‐brushing within 120 min [10, 11]. This dynamic is influenced by factors such as the form and solubility of fluoride in the formulation, post‐brushing rinsing practices, and salivary flow rate [10, 12]. During sleep, when salivary flow is markedly reduced, fluoride availability in the oral cavity may be significantly altered [13].

Dentifrices containing 1450 ppm F represent the standard formulation recommended for daily use by the general population, providing effective caries prevention through regular topical fluoride exposure [14]. In contrast, high‐fluoride dentifrices (5000 ppm F) are indicated for individuals at elevated risk of caries, such as those with reduced salivary flow, orthodontic appliances, root exposure, or a history of active carious lesions [15]. Indeed, high‐fluoride dentifrices (5000 ppm F) have been shown to increase fluoride levels in saliva, biofilm fluid, and solids; reduce mineral loss and lesion depth in demineralized dentin; reverse non‐cavitated lesions; and potentially promote greater calcium fluoride deposition compared to regular dentifrices containing 1000–1500 ppm F) [9, 16, 17].

Despite the proven clinical benefits of high‐fluoride dentifrices, particularly in high‐risk groups, limited data exist regarding their pharmacokinetics under different physiological conditions, such as during nocturnal periods with reduced salivary flow. Comparing these two formulations under both diurnal and nocturnal conditions offers valuable insights into their respective capacities to maintain therapeutic fluoride levels in the oral cavity over time, which is crucial for optimizing individualized caries prevention strategies. Therefore, this study aimed to evaluate the salivary fluoride bioavailability during diurnal and nocturnal periods following a single brushing with a high‐fluoride dentifrice, and to compare it with the fluoride bioavailability achieved using a regular 1450 ppm F dentifrice.

## MATERIAL AND METHODS

### Ethical considerations

This study was approved by the Research Ethics Committee of the Federal University of Piaui (protocol number 483,913). All participants provided written informed consent. The research was conducted in accordance with Resolution No. 466 of the Brazilian National Health Council, the guidelines of the Ministry of Health, and the principles outlined in the Declaration of Helsinki. This study is reported according to the CONSORT (Consolidated Standards of Reporting Trials) guidelines.

### Study design and participants

A randomized short‐term clinical trial with a crossover design was conducted with 15 healthy adult participants (6 males and 9 females; mean age: 24.4 ± 2.5 years), all residing in a municipality with fluoridated public water supplies (0.6–0.8 ppm F), which were submitted at four experimental conditions derived from the combination of two dentifrice formulations (1450 and 5000 ppm F) and two brushing periods (diurnal and nocturnal). Participants were allocated to treatment sequences according to a Williams Latin square design. Specifically, each participant completed the four experimental phases in a unique order, and all possible treatment sequences were arranged so that each treatment appeared once in each period position, and each treatment was preceded equally often by each of the other treatments.

Eligible participants were adults in good general and oral health (i.e., free from chronic systemic diseases) with a normal stimulated salivary flow rate ≥2 mL in 2 min. Exclusion criteria were smoking, active caries lesions, periodontal disease, orthodontic appliances, cardiovascular disease, pregnancy, lactation, and the use of medications known to reduce salivary flow (e.g., anticonvulsants, tricyclic antidepressants, and hormonal contraceptives) and the use of antibiotics for the last month before study. All participants underwent a dental examination and completed a questionnaire addressing general health status and medication use.

The sample size was calculated based on previous studies [18] using a similar experimental design. The parameters considered included four experimental groups, an estimated standard deviation of the salivary fluoride concentration of 0.55 µg F/mL, a minimum detectable salivary fluoride concentration difference of 0.45 µg F/mL between groups, an alpha error of 5%, and a beta error of 20%. The calculated sample size was 13 participants; anticipating a potential 20% dropout rate, the final sample size was set at 15.

**FIGURE 1:**
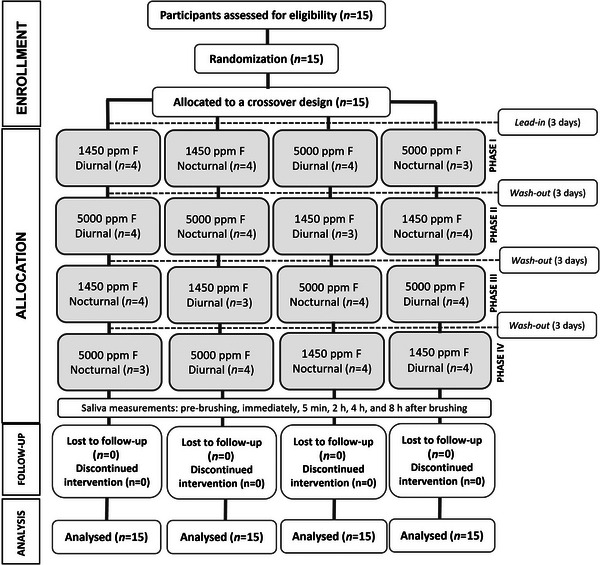
The CONSORT flow diagram of the study.

Each participant underwent four experimental phases in which they randomly performed a single brushing with one of the two dentifrices (1450 or 5000 ppm F) during either the diurnal (8:00 a.m. to 4:00 p.m.) or nocturnal (10:00 p.m. to 6:00 a.m.) periods (Figure 1). They were advised to avoid fluoride‐rich foods (e.g., tea and fish) during the saliva collection period but were not restricted from drinking water. Due to the crossover design of the study, no dietary restrictions were imposed. A 3‐day lead‐in and washout period with fluoride‐free dentifrice was applied before and between each experimental phase. Randomization of participants to sequences was performed prior to the start of the study by an investigator not involved in data collection.

### Dentifrices

Two commercially available dentifrices with different fluoride concentrations were used in the experimental phases: one regular dentifrice containing 1450 ppm F and one high‐fluoride dentifrice containing 5000 ppm F. Both products used sodium fluoride (NaF) as the fluoride source, silica as abrasive, and were manufactured by the same company (Table 1). To ensure blinding, the dentifrices were dispensed into identical plastic tubes labeled only with a code.

**TABLE 1:** Composition of the dentifrices used in the study.

Dentifrice brand manufacturer	Fluoride concentration (ppm F) declared^a^	Soluble fluoride concentration (ppm F) observed (SD)^b^	Fluoride salt form^a^	Ingredients^a^
Colgate total 12, Colgate‐Palmolive	1450	1489 (37)	NaF	Glycerin, water, hydrated silica, sodium lauryl sulfate, arginine, flavor, zinc oxide, cellulose gum, poloxamer 407, tetrasodium pyrophosphate, zinc citrate, benzyl alcohol, cocamidopropyl betaine, sodium saccharin, xanthan gum, phosphoric acid, sucralose CI 77891, titanium dioxide (CI 77891).
OrthoGard, Colgate‐Palmolive	5000	5055 (120)	NaF	Water, sorbitol, hydrated silica, PEG‐12, tetrapotassium pyrophosphate, sodium lauryl sulfate, flavor, xanthan gum, sodium benzoate, sodium saccharin, FD&C blue (CI 42090)

Abbreviation: NaF, sodium fluoride; SD, standard deviation.

^a^ Informed by the manufacturer.

^b^ *n* = 5.

### Determination of total soluble fluoride concentration in dentifrices

To determine the total soluble fluoride concentration in the dentifrices, approximately 1 g of each dentifrice was weighed using an analytical balance and homogenized with 30 mL of deionized water. A 1 mL aliquot of this suspension was mixed with 1 mL of TISAB II and analyzed using a fluoride ion‐selective electrode (Analyser 18AF) calibrated with standard fluoride solutions ranging from 0.5 to 64 µg F/mL. Measurements were performed in quintuplicate and expressed in µg F/mL (Table 1).

### Saliva collection

Saliva collection was performed after a single brushing episode with the assigned fluoridated dentifrice during either the diurnal or nocturnal period, with a minimum interval of 12 h since the last exposure to a fluoride‐free dentifrice. During washout periods, participants resumed their usual brushing frequency using only the fluoride‐free dentifrice. Participants were provided with kits containing a toothbrush, saliva collection containers, a disposable cup of standardized volume (15 mL) for post‐brushing rinsing, a fluoride‐free dentifrice (placebo), and the test dentifrices assigned to each study phase. They received both verbal and written instructions to brush their teeth for one minute using approximately 1 g of dentifrice (corresponding to the full length of the brush bristles). Two milliliters of unstimulated saliva were collected in a prelabeled test tube before (pre‐brushing) and immediately after brushing and water rinse and at times 5 min, 2 h, 4 h, and 8 h after brushing. The water rinse was performed with 15 mL of tap water for 5 s. During the night, in the saliva‐collection period of 2 and 4 h, the participants woke up, performed the collections, and returned to bed. All saliva samples were immediately stored in the participants’ household freezers after collection. On the following day, samples were retrieved by the responsible researcher and transferred to the laboratory, where they were kept at −20°C until analysis.

### Determination of fluoride concentration in saliva

Fluoride concentrations in saliva were determined using a fluoride‐specific electrode (Analyser 18AF‐001, Analyser Analytical Instrumentation) coupled to an ion analyzer (Orion EA‐740, Thermo Scientific). One‐milliliter aliquots of saliva were centrifuged at 5000 g for 3 min (Kasvi). Then, 0.5 mL of the supernatant was mixed with an equal volume of TISAB II buffer. Fluoride concentrations were calculated using a linear regression calibration curve obtained from standard fluoride solutions ranging from 0.0625 to 32 µg F/mL, prepared under the same conditions as the saliva samples. Samples exceeding the calibration range were appropriately diluted to fall within the range of the standard curve.

### Area under the curve calculation

The area under the curve (AUC) of salivary fluoride concentration over time was calculated using graphpad prism 9.0 (GraphPad Software). Calculations were performed using the trapezoidal method, which assumes linear changes between consecutive data points and sums the area of trapezoids formed under the curve. The actual time intervals between collections (0, 5, 120, 240, and 480 min) were used in the analysis to ensure accurate estimation of fluoride bioavailability.

### Statistical analysis

Salivary fluoride concentration data normality was assessed using Shapiro–Wilk test, which indicated non‐normal distribution of the data, except for AUC data, which presented normal distribution after log transformation of fluoride concentration data. Therefore, statistical comparisons between dentifrices (1450 and 5000 ppm F) and between periods (diurnal and nocturnal) were performed using the Wilcoxon matched‐pairs test. Post‐brushing salivary fluoride concentrations at different time points were compared to pre‐brushing using the Kruskal–Wallis test, followed by Dunn's multiple comparisons post hoc test. The AUC was analyzed using Anova followed by Tukey's post hoc test, with the significance level set at 5%. Descriptive statistics are presented in graphs and tables. All statistical analyses were conducted using graphpad prism version 9.02 (GraphPad Software).

## RESULTS

Table 2 presents the median and the interquartile range of salivary fluoride concentrations (µg F/mL) following the use of dentifrices containing 1450 and 5000 ppm F during the diurnal and nocturnal periods. During the diurnal period, statistically significantly higher fluoride concentrations were observed at 0 min, 5 min, and 2 h after brushing with the 5000 ppm F dentifrice than with the 1450 ppm F dentifrice (*p* < 0.05). In the nocturnal period, salivary fluoride concentrations were statistically significantly higher for the 5000 ppm F dentifrice at all time points except pre‐brushing (*p* < 0.05). Pre‐brushing salivary fluoride concentrations did not differ across experimental conditions (*p* > 0.05), indicating comparable starting levels before brushing and supporting the effectiveness of the fluoride‐free washout period in minimizing residual fluoride carry‐over between phases. However, for the 1450 ppm F dentifrice, significantly higher fluoride levels were detected during the nocturnal period than during the diurnal period at 0 min, 5 min, 2 h, and 4 h (*p* < 0.05). For the 5000 ppm F dentifrice, higher salivary fluoride concentrations were also found during the nocturnal period than during the diurnal period at 2, 4, and 8 h post‐brushing (*p* < 0.05).

**TABLE 2:** Median (IQR) of salivary fluoride concentration (µg F/mL) according to the dentifrices (1450 or 5000 ppm F) and periods (diurnal or nocturnal).

Time	1450 ppm F		5000 ppm F	
	Diurnal	Nocturnal	Diurnal	Nocturnal
Pre‐brushing	0.06 (0.03)^aA^	0.06 (0.06)^aA^	0.06 (0.02)^aA^	0.06 (0.02)^aA^
Immediately	7.05 (9.57)^aA^	13.48 (18.37)^bA^	40.11 (92.20)^aB^	41.53 (117.28)^aB^
5 min	1.11 (4.75)^aA^	2.41 (9.50)^bA^	7.99 (20.97)^aB^	8.06 (31.06)^aB^
2 h	0.09 (0.07)^aA^	0.17 (0.28)^bA^	0.15 (0.15)^aB^	0.39 (0.48)^bB^
4 h	0.06 (0.03)^aA^	0.08 (0.08)^bA^	0.10 (0.08)^aA^	0.20 (0.33)^bB^
8 h	0.07 (0.04)^aA^	0.07 (0.03)^aA^	0.06 (0.05)^aA^	0.12 (0.14)^bB^

*Note*: Different lowercase letters indicate a statistical difference between the periods within the same dentifrice, while different capital letters indicate a statistical difference between the dentifrices within the same period (*p* < 0.05) by the Wilcoxon matched‐pairs test.

Abbreviation: IQR, interquartile range.

**FIGURE 2:**
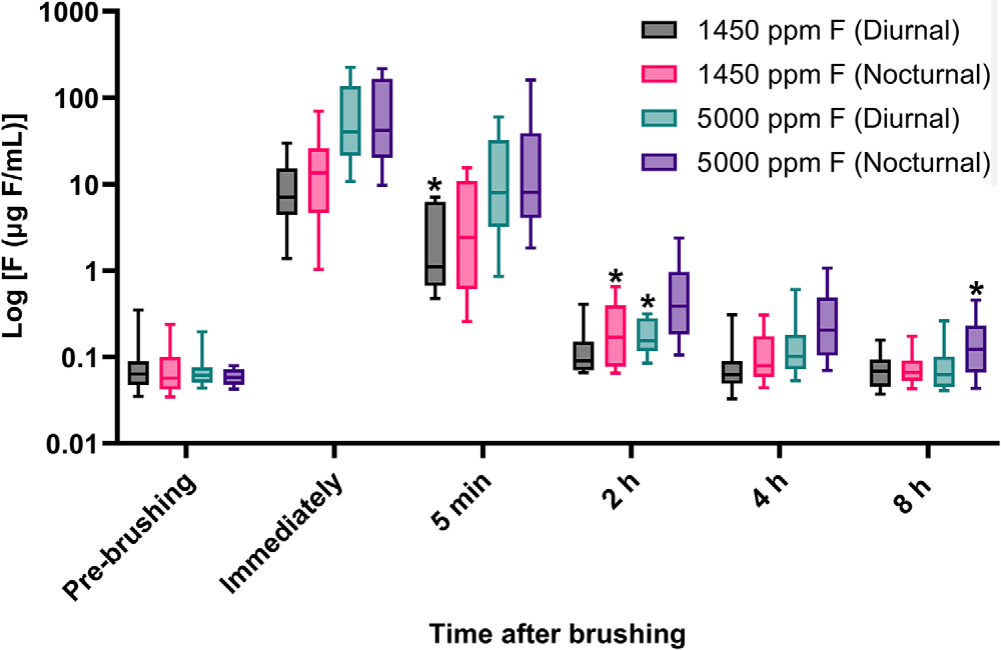
Kinetics (mean ± SD, *n* = 15) of log‐transformed salivary fluoride concentration (log_10_ µg F/mL) according to time after brushing with different dentifrices and periods. The maximum concentration of fluoride is reached immediately after brushing for all groups. The asterisks show the time point at each salivary fluoride concentration reaches the pre‐brushing values by Dunn's test (*p* < 0.05). Time points on the *X*‐axis are displayed at equal intervals, and *Y*‐axis values were log‐transformed to facilitate visualization. SD, standard deviation.

Figure 2 displays the kinetic curves of salivary fluoride concentration (µg F/mL) over time following brushing with both dentifrices during diurnal and nocturnal periods. For all groups, the peak fluoride concentration occurred immediately after brushing, followed by a progressive decline. The highest salivary fluoride concentrations were recorded after using the 5000 ppm F dentifrice during the nocturnal period, with elevated levels persisting for up to 8 h (*p* < 0.05). This figure also illustrates the time required for salivary fluoride levels to return to pre‐brushing. For the 1450 ppm F dentifrice, fluoride concentrations returned to pre‐brushing after 5 min (diurnal) and 2 h (nocturnal). In contrast, for the 5000 ppm F dentifrice, pre‐brushing levels were reached at 2 h (diurnal) and 8 h (nocturnal) post‐brushing (*p* < 0.05).

**FIGURE 3:**
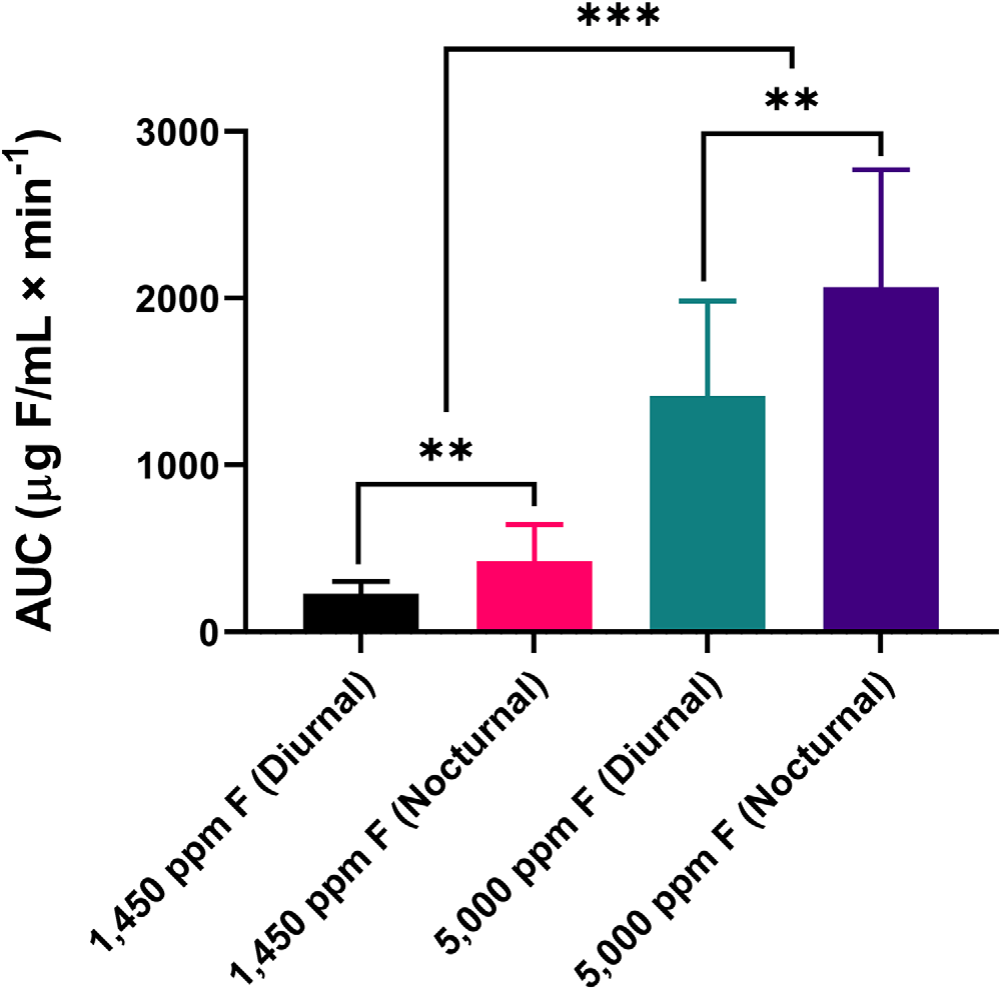
Mean of the area under the curve (AUC) of salivary fluoride concentration as a function of time (µg F/mL min^−1^) according to treatments (*n* = 15). ^**^indicate the statistical difference between the same dentifrice in different periods (*p* < 0.05). ^***^indicate the statistical difference between different dentifrices within the same period (*p* < 0.05). Vertical bars indicate the standard deviation.

Figure 3 shows the AUC for salivary fluoride concentration over time. Statistically significant differences were found between the diurnal and nocturnal periods, regardless of the dentifrice used (*p* < 0.05). Additionally, the 5000 ppm F dentifrice showed significantly higher AUC values than the 1450 ppm F dentifrice in both periods (*p* < 0.05), indicating greater overall fluoride bioavailability, especially during the nocturnal period.

## DISCUSSION

In this study, both dentifrices increased salivary fluoride concentrations following brushing; however, significantly higher concentrations were observed after using the high‐fluoride (5000 ppm F) dentifrice. These findings align with previous reports demonstrating greater fluoride bioavailability with high‐fluoride formulations [11, 19, 20]. Notably, this is the first study to compare the salivary fluoride kinetics of regular and high‐fluoride dentifrices across both diurnal and nocturnal periods, providing new insights into circadian effects on fluoride retention in the oral cavity.

The differences in salivary fluoride concentrations observed between the regular and high‐fluoride dentifrices support the dose‐response relationship of fluoride and confirm that saliva is a sensitive indicator capable of detecting variations between treatments and across time points following dentifrice use [18]. These results can be attributed in part to the formulations of the products. Both NaF/silica‐based dentifrices demonstrated total soluble fluoride concentrations consistent with their labeled content, ensuring effective delivery of bioavailable fluoride [21], as fluoride must remain chemically soluble within the formulation to exert its preventive effects [1]. Nevertheless, it should be acknowledged that dentifrices differ not only in fluoride concentration but also in other excipients and active compounds such as abrasives, humectants, and detergents, which may influence fluoride availability, salivary clearance, and even interaction with oral surfaces. Although both formulations tested here were silica‐based, such compositional variations may have contributed to the observed differences in peak salivary fluoride concentrations and in the rate and duration of fluoride retention over time, particularly under nocturnal conditions, and warrant further investigation.

The analysis of the AUC for salivary fluoride confirmed that the 5000 ppm F dentifrice maintained significantly higher fluoride levels over time compared to the regular formulation, in both diurnal and nocturnal periods. Additionally, peak salivary fluoride concentrations were higher for participants using the high‐fluoride dentifrice. These findings underscore the superior potential of high‐fluoride products to protect dental substrates, especially root surfaces, which are more susceptible to caries and require higher fluoride concentrations for effective remineralization [22]. This is clinically relevant for high‐risk populations, such as individuals with active caries, reduced salivary flow, or those undergoing orthodontic treatment, where high‐fluoride dentifrices have demonstrated efficacy in reducing demineralization around brackets [17, 23, 24].

The nocturnal period warrants particular consideration due to the physiological reduction in salivary flow during sleep, which limits both mechanical clearance and buffering capacity in the oral cavity. While both dentifrices were found to elevate salivary fluoride levels at night, only the high‐fluoride dentifrice sustained elevated concentrations up to 8 h post‐brushing. In contrast, fluoride concentrations returned to pre‐brushing within 2 h following the use of the regular formulation. This extended fluoride bioavailability during nocturnal hyposalivation may provide crucial protection when the risk of demineralization is heightened due to reduced salivary defense mechanisms [13].

The clinical implication of these findings is the reinforcement of nightly toothbrushing with fluoridated dentifrices, particularly those with high fluoride concentration. During sleep, oral clearance and the natural protective mechanisms against acid challenges are impaired [13]. Under these conditions, residual fermentable carbohydrates in dental biofilm or late‐evening acidic exposures may lead to prolonged low pH episodes, favoring demineralization. The prolonged fluoride availability provided by the 5000 ppm F formulation before bedtime may therefore enhance protection against nocturnal demineralization. In addition, extended intraoral fluoride retention favors the formation of fluoride reservoirs [7, 9], which can gradually release ions into saliva and biofilm, sustaining cariostatic activity throughout the night.

It is important to address some limitations of the present study. Although the experimental design involved awakening participants during the night for saliva collection, allowing accurate and consistent sampling, this simulated sleep condition may have temporarily increased salivary flow compared to continuous sleep, possibly underestimating the extent of fluoride retention under real nocturnal hypofunction. Additionally, interindividual differences in brushing technique, dentifrice foam removal, and post‐brushing rinsing, despite instructions and standardization and the crossover design, may have contributed to variability in salivary fluoride levels. These should be considered when generalizing the findings.

Another methodological limitation concerns the use of TISAB II in fluoride analysis. Although there is a recommendation to use TISAB III in samples with low concentrations of F, there is no consensus in the literature on which TISAB to use to buffer saliva samples to determine F concentration, since some used TISAB II [6, 18] and others used TISAB III [9, 20]. Although TISAB II has been routinely employed in our laboratory and is well‐documented in the literature, it may present reduced sensitivity for detecting very low fluoride concentrations, particularly at later collection times when salivary fluoride levels approach pre‐brushing. This methodological choice, even though internally validated and consistent with previous studies, could have influenced the accuracy of results under critical low‐fluoride conditions.

In conclusion, the high‐fluoride dentifrice significantly enhanced salivary fluoride bioavailability compared to the regular formulation, during both daytime and nighttime use. These results support its clinical indication for managing dental caries, particularly in individuals at elevated risk. The prolonged fluoride retention observed during the night further reinforces the recommendation of its use before bedtime as part of a targeted caries‐prevention strategy, particularly when salivary protection is naturally reduced.

## AUTHOR CONTRIBUTIONS

**Conceptualization**: Vale G. **Investigation**: Carvalho G. **Methodology**: Carvalho G, Santana G, Macena N. **Supervision**: Vale G. **Writing—original draft**: Carvalho G, Vale G. **Writing—review and editing**: Vale G.

## CONFLICT OF INTEREST STATEMENT

The authors declare no conflicts of interest.

## Data Availability

All data generated or analyzed during this study are included in this article. Further inquiries can be directed to the corresponding author.

## References

[1] Tenuta LM , Cury JA . Fluoride: its role in dentistry. Braz Oral Res. 2010;24(Suppl 1):9–17. 10.1590/s1806-83242010000500003 20857070

[2] Featherstone JD . Prevention and reversal of dental caries: role of low level fluoride. Community Dent Oral Epidemiol. 1999;27:31–40. 10.1111/j.1600-0528.1999.tb01989.x 10086924

[3] Cury JA , Tenuta LM . How to maintain a cariostatic fluoride concentration in the oral environment. Adv Dent Res. 2008;20:13–6. 10.1177/154407370802000104 18694871

[4] Tenuta LM , Zamataro CB , Del B Cury AA , Tabchoury CP , Cury JA . Mechanism of fluoride dentifrice effect on enamel demineralization. Caries Res. 2009;43:278–85. 10.1159/000217860 19439949

[5] Bratthall D , Hänsel‐Petersson G , Sundberg H . Reasons for the caries decline: what do the experts believe? Eur J Oral Sci. 1996;104:416–22; discussion 423–5, 430–2. 10.1111/j.1600-0722.1996.tb00104.x 8930592

[6] Naumova EA , Staiger M , Kouji O , Modric J , Pierchalla T , Rybka M , et al. Randomized investigation of the bioavailability of fluoride in saliva after administration of sodium fluoride, amine fluoride and fluoride containing bioactive glass dentifrices. BMC Oral Health. 2019;19:119. 10.1186/s12903-019-0805-6 31215467 PMC6582593

[7] Duckworth RM , Morgan SN . Oral fluoride retention after use of fluoride dentifrices. Caries Res. 1991;25:123–9. 10.1159/000261354 2059973

[8] Duckworth RM , Jones S . On the relationship between the rate of salivary flow and salivary fluoride clearance. Caries Res. 2015;49:141–6. 10.1159/000365949 25634162

[9] Staun Larsen L , Baelum V , Richards A , Nyvad B . Fluoride in saliva and oral mucosa after brushing with 1,450 or 5,000 ppm fluoride toothpaste. Caries Res. 2019;53:675–81. 10.1159/000501264 31307037

[10] Naumova EA , Kuehnl P , Hertenstein P , Markovic L , Jordan RA , Gaengler P , et al. Fluoride bioavailability in saliva and plaque. BMC Oral Health. 2012;12:3. 10.1186/1472-6831-12-3 22230722 PMC3295678

[11] Pessan JP , Conceição JM , Grizzo LT , Székely M , Fazakas Z , Buzalaf MA . Intraoral fluoride levels after use of regular and high‐fluoride dentifrices. Clin Oral Investig. 2015;19:955–8. 10.1007/s00784-015-1426-3 25677244

[12] Issa AI , Toumba KJ . Oral fluoride retention in saliva following toothbrushing with child and adult dentifrices with and without water rinsing. Caries Res. 2004;38:15–9. 10.1159/000073915 14684972

[13] Dawes C . Circadian rhythms in human salivary flow rate and composition. J Physiol. 1972;220:529–45. 10.1113/jphysiol.1972.sp009721 5016036 PMC1331668

[14] Walsh T , Worthington HV , Glenny AM , Marinho VC , Jeroncic A . Fluoride toothpastes of different concentrations for preventing dental caries. Cochrane Database Syst Rev. 2019;3:CD007868. 10.1002/14651858.CD007868.pub3 30829399 PMC6398117

[15] Ekstrand KR . High fluoride dentifrices for elderly and vulnerable adults: does it work and if so, then why? Caries Res. 2016;50(Suppl 1):15–21. 10.1159/000443021 27101401

[16] Ekstrand KR , Ekstrand ML , Lykkeaa J , Bardow A , Twetman S . Whole‐saliva fluoride levels and saturation indices in 65+ elderly during use of four different toothpaste regimens. Caries Res. 2015;49:489–98. 10.1159/000434730 26278523

[17] Al‐Mulla A , Karlsson L , Kharsa S , Kjellberg H , Birkhed D . Combination of high‐fluoride toothpaste and no post‐brushing water rinsing on enamel demineralization using an in‐situ caries model with orthodontic bands. Acta Odontol Scand. 2010;68:323–8. 10.3109/00016357.2010.512863 20831358

[18] Macena NS , Santana GB , Carvalho GAO , Vale GC . Salivary fluoride bioavailability after use of high‐fluoride dentifrices with different compositions: a short‐term randomized clinical trial. Int J Dent Hyg. 2024;22:514–20. 10.1111/idh.12668 36628516

[19] Downey D , Dennison J , Eckert GJ , Flannagan SE , Neiva GF , Yaman P , et al. Fluoride levels in unstimulated whole saliva following clinical application of different 5% NaF varnishes. Caries Res. 2018;52:431–8. 10.1159/000485981 29614502

[20] Staun Larsen L , Baelum V , Tenuta LMA , Richards A , Nyvad B . Fluoride in saliva and dental biofilm after 1500 and 5000 ppm fluoride exposure. Clin Oral Investig. 2018;22:1123–9. 10.1007/s00784-017-2195-y 28865065

[21] Tenuta LM , Cury JA . Laboratory and human studies to estimate anticaries efficacy of fluoride toothpastes. Monogr Oral Sci. 2013;23:108–24. 10.1159/000350479 23817064

[22] Hoppenbrouwers PM , Driessens FC , Borggreven JM . The mineral solubility of human tooth roots. Arch Oral Biol. 1987;32:319–22. 10.1016/0003-9969(87)90085-9 2821975

[23] Sonesson M , Twetman S , Bondemark L . Effectiveness of high‐fluoride toothpaste on enamel demineralization during orthodontic treatment‐a multicenter randomized controlled trial. Eur J Orthod. 2014;36:678–82. 10.1093/ejo/cjt096 24375756

[24] Ferreira RS , Ricomini‐Filho AP , Tabchoury CP , Vale GC . Effect of high‐fluoride dentifrice and bracket bonding composite material on enamel demineralization in situ. Clin Oral Investig. 2020;24:3105–12. 10.1007/s00784-019-03182-7 31897706

